# Dynamic molecular oxygen production in cometary comae

**DOI:** 10.1038/ncomms15298

**Published:** 2017-05-08

**Authors:** Yunxi Yao, Konstantinos P. Giapis

**Affiliations:** 1 Division of Chemistry and Chemical Engineering, California Institute of Technology, Pasadena, California 91125, USA

## Abstract

Abundant molecular oxygen was discovered in the coma of comet 67P/Churyumov–Gerasimenko. Its origin was ascribed to primordial gaseous O_2_ incorporated into the nucleus during the comet's formation. This thesis was put forward after discounting several O_2_ production mechanisms in comets, including photolysis and radiolysis of water, solar wind–surface interactions and gas-phase collisions. Here we report an original Eley–Rideal reaction mechanism, which permits direct O_2_ formation in single collisions of energetic water ions with oxidized cometary surface analogues. The reaction proceeds by H_2_O^+^ abstracting a surface O-atom, then forming an excited precursor state, which dissociates to produce O_2_^−^. Subsequent photo-detachment leads to molecular O_2_, whose presence in the coma may thus be linked directly to water molecules and their interaction with the solar wind. This abiotic O_2_ production mechanism is consistent with reported trends in the 67P coma and raises awareness of the role of energetic negative ions in comets.

## Abstract (web summary)

Abundant molecular oxygen was discovered recently in the coma of comet 67P, thought to be of primordial origin. Here, the authors propose a dynamic reaction mechanism for cometary comae, which produces O_2_ directly in single collisions of energetic water ions with oxidized cometary surface analogues.

Although oxygen is the third most abundant element in the universe, its molecular form (dioxygen, O_2_) is very rare. Molecular oxygen has only been detected in two interstellar clouds, the Orion Nebula^1^ and the *ρ* Oph A dense core^2^. In contrast to Earth, where oxygenic photosynthesis has made O_2_ abundant, only tenuous amounts of dioxygen are found elsewhere in our solar system, for example, in the moons of Jupiter^3^, Saturn^4^ and on Mars^5^. Remarkably, molecular oxygen was detected recently in the coma of comet 67P/Churyumov–Gerasimenko by the Rosetta spacecraft, with local O_2_ abundances ranging between 1 and 10% relative to water^6^. The O_2_ was proposed to be of primordial origin in conflict with Solar System formation theories^6^.

Understanding the origin of molecular oxygen in space is important for the evolution of the Universe and the origin of life on Earth^7^,^8^,^9^. In fact, molecular oxygen in abundance has been suggested as a promising biomarker^10^. Interstellar and cometary oxygen is strongly bound chemically to other elements in compounds, such as H_2_O, CO_2_, CO, silicates and metal oxides. Release of O_2_ from these reservoirs is practically difficult and energetically very expensive. Energetic particles, such as photons, electrons and ions, exist in abundance in astrophysical environments and can initiate dissociation reactions that ultimately produce O_2_. Apropos, photolysis and radiolysis of water, solar wind interactions with the nucleus surface, and gas-phase collisions in the coma have been considered but found deficient in explaining the origin of O_2_ in the coma of comet 67P (ref. 6). Despite their cometary abundance, energetic molecular ions such as H_2_O^+^ and H_3_O^+^ have not been discussed in the context of O_2_ production.

When energetic molecules collide with surfaces, they may dissociate promptly, undergo electronic excitation or participate in surface reactions. The latter include Eley–Rideal (ER) reactions, where energetic projectiles collide with surfaces and react with adsorbates to produce projectile-adsorbate molecules without equilibration with the surface^11^. This dynamic process is driven by the projectile energy, a large fraction of which is carried away by the product molecule. Despite the implied similarity, the ER reaction process is different from sputtering (physical ejection of surface matter): a targeted new bond is formed, typically at hyperthermal incidence energies (10–200 eV). Notably, ER reactions have no surface temperature dependence and thus could be important in cometary environments during active periods, when energetic molecular ions are generated through interactions with the solar wind. Indeed, ‘accelerated' water ions have been discovered in the inner coma of comet 67P (refs 12, 13). These ions possess kinetic energy between 120 and 800 eV, and impact and sputter the nucleus surface at fluxes comparable to the typical solar wind flux^13^. The outer crust of the 67P nucleus facing the Sun is dehydrated^14^, thus exposing mineral surfaces to the ions. Minerals found on the comet, such as olivine and pyroxene silicates^15^,^16^ and Fe/Ni oxides^17^, are oxidized offering another potential source of oxygen. Thus, collisions of energetic water ions with oxidized minerals on the nucleus surface are probable, with several possible outcomes: (1) collision-induced dissociation (CID) of H_2_O^+^ produces atomic O, atomic H and OH radicals and ions; (2) physical sputtering ejects mineral constituents, including metal and oxygen atoms; and (3) collisional excitation of H_2_O^+^ drives an intramolecular water-splitting reaction^18^, producing molecular H_2_ directly. But there is also a surprising forth outcome.

We have discovered and verified in laboratory experiments that energetic water ions can also participate in ER abstraction reactions on oxidized surfaces to directly form molecular O_2_ anions. When oxygen is abstracted from such surfaces, it is readily replenished by (a) O fragments from the CID of H_2_O^+^ and (b) freshly exposed O atoms from physical sputtering of the mineral surface by H_2_O^+^. Thus, the ER reaction mechanism may generate O_2_^−^ continually on the cometary surface, which is emitted into the coma with high kinetic energy.

## Results

### O_2_^−^ and HO_2_^−^ production on cometary surface analogues

**Figure 1:**
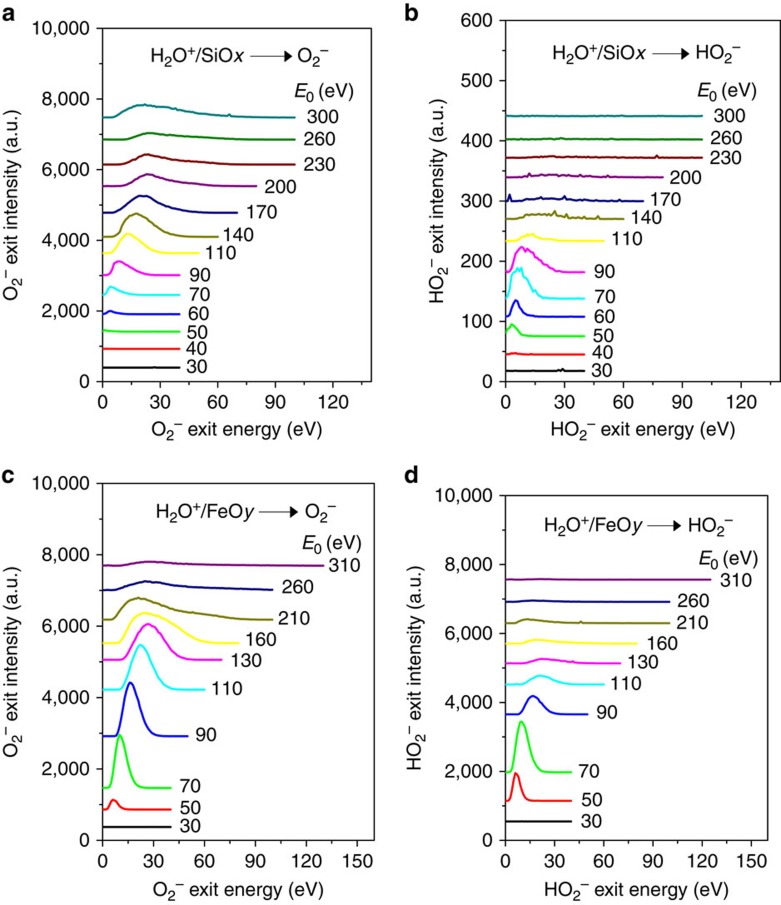
Production of O_2_^−^ and HO_2_^−^ from energetic H_2_O^+^ bombardment of oxides. Energy distributions of (**a**,**c**) O_2_^−^ and (**b**,**d**) HO_2_^−^ scattered from native Si oxide (**a**,**b**) and Fe oxide (**c**,**d**) following bombardment by H_2_O^+^ at various incidence energies. Physical sputtering contributions to the O_2_^−^ signal become visible at high energies (*E*_0_>250 eV for SiO_*x*_, *E*_0_>150 eV for FeO_*y*_), when the dynamic O_2_^−^ peak dies out.

We first demonstrate the production of molecular oxygen and hydroperoxyl radicals, detected as anions, from H_2_O^+^ ions bombarding Si, and Fe targets. These surfaces are covered with amorphous native oxide (hereafter SiO_*x*_ and FeO_*y*_), selected as analogues of two inorganic minerals commonly found on comets. Scattering on these surfaces produces multiple species from dissociation and physical sputtering, but also from direct reactions. Negative ion formation (Supplementary Figs 1 and 2) is of particular interest, since surface scattering has not been considered before as a production mechanism in cometary environments. Figure 1 shows energy distributions for O_2_^−^ and HO_2_^−^, scattered off from SiO_*x*_ and FeO_*y*_ as a function of the H_2_O^+^ incidence energy (*E*_0_). For both surfaces, O_2_^−^ signal appears above *E*_0_∼50–60 eV. The peak position shifts to higher energy and the peak intensity goes through a maximum with increasing incidence energy. The scattering signal dies out above ∼200 eV (Fig. 1a,c). The same is true for HO_2_^−^, though this peak appears earlier and dies out sooner than O_2_^−^ (Fig. 1b,d). These trends will be understood below considering that HO_2_^−^ is a precursor to O_2_^−^. Are these anions sputtering products? Experiments with Ne^+^ beams scattering on SiO_*x*_ and FeO_*y*_ surfaces indicate that O_2_^−^ is produced with intensity of ∼1–2% of that seen for H_2_O^+^ at *E*_0_=110 eV. Increasing the energy to *E*_0_=310 eV, causes the O_2_^−^ signal to increase to 3% and 25% for SiO_*x*_ and FeO_*y*_, respectively, compared to H_2_O^+^ at the same energy (Supplementary Fig. 3). Under ^18^O_2_ dosing, the sputtering contribution to O_2_^−^ signal increases to ∼15% for H_2_O^+^/FeO_*y*_, while undetectable for H_2_O^+^/SiO_*x*_ at *E*_0_=110 eV (Supplementary Fig. 4; Supplementary Table 1). Of course, sputtering is expected to increase for incidence energies above 300 eV, but the focus here is on much lower energies. With sputtering ruled out, the origin of O_2_^−^ is puzzling.

### Isotopic water scattering experiments on a model Pt surface

Owing to the light atomic mass of Fe and Si, H_2_O^+^ scattering on cometary surface analogues produces dynamic O_2_^−^ peaks with energies over a limited range, with little separation from sputtering signal, thus hindering kinematic analysis. Collisional energy transfer is reduced on Pt, yielding larger spread in exit energies and much reduced overlap with sputtering peaks. Of course, the Pt surface needs to be oxidized first to render O-atom abstraction experiments possible. As mentioned above, CID of water ions is expected to supply O atoms to the Pt surface (proven below). However, neither H_2_O^+^ nor D_2_O^+^ ions, scattering on clean Pt, produce any discernible O_2_^−^ signal. This is likely due to molecular hydrogen, present in the ultrahigh vacuum background or formed *in situ* by an intramolecular water-splitting reaction^18^, which scavenges O atoms from the Pt surface. The O-atom surface coverage must therefore be increased to overcome this problem, which is achieved readily by dosing the surface *in situ* with O_2_ gas. Molecular O_2_ dissociates spontaneously on Pt at room temperature^19^, covering the surface with adsorbed O atoms—denoted hereafter as Pt(O).

**Figure 2:**
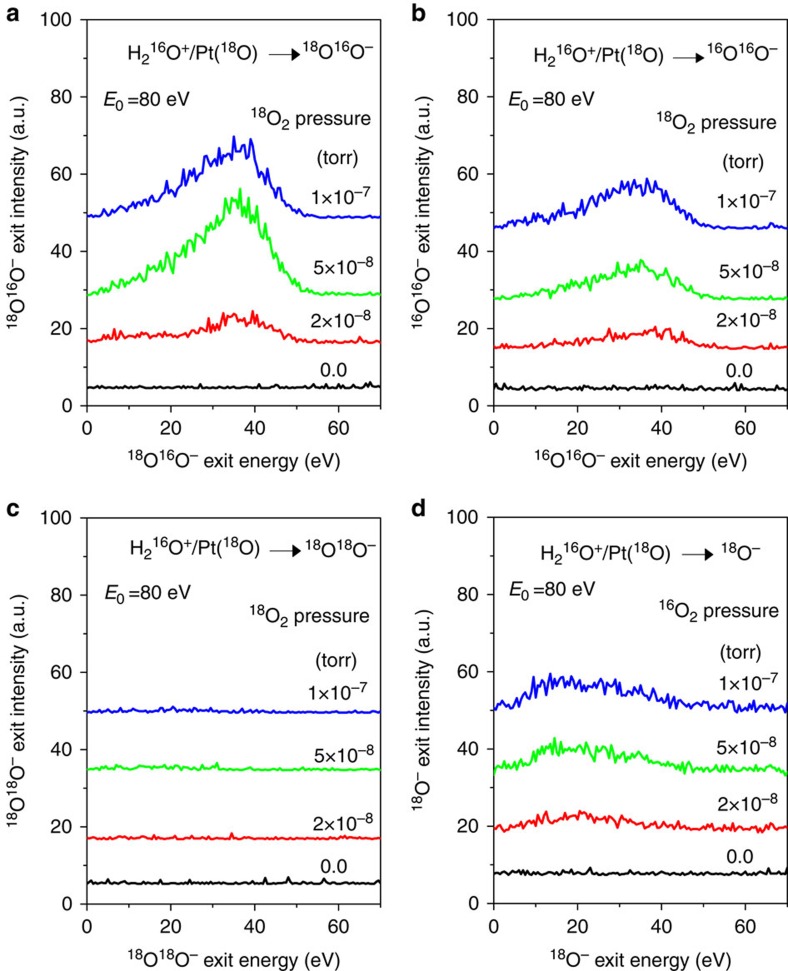
Isotopic dosing experiments of H_2_O^+^ scattering on Pt covered with ^18^O atoms. Energy distributions of ion exits of (**a**) ^18^O^16^O^−^, (**b**) ^16^O^16^O^−^, (**c**) ^18^O^18^O^−^ and (**d**) ^18^O^−^ from H_2_O^+^ scattering on Pt at various ^18^O_2_ exposure pressures, as annotated. The detection of ^18^O^16^O^−^ in **a** indicates fast O_2_^−^ formation between ^16^O-atoms from H_2_^16^O^+^ and ^18^O-atoms adsorbed on the Pt surface. The absence of ^18^O^18^O^−^ in **c** proves that fast O_2_^−^ may not originate in the gas phase or from surface sputtering. The ^16^O^16^O^−^ formation in **b** is due to the oxygen deposition on the Pt surface from collision-induced dissociation of H_2_^16^O^+^ (see text). The appearance of ^18^O^−^ sputtering peak ∼25 eV in **d** confirms that the surface is covered by ^18^O atoms, produced by *in situ*^18^O_2_ exposure.

Upon exposure of the Pt surface to ^16^O_2_ gas, a strong dynamic ^16^O_2_^−^ peak appears for both H_2_^16^O^+^ and D_2_^16^O^+^ beams (Supplementary Fig. 5). What is the origin of the observed ^16^O_2_^−^? Isotopic ^18^O_2_ dosing experiments on Pt, bombarded with normal H_2_^16^O^+^ projectiles, can help distinguish possible contributions from sputtering or gas-phase collisions. Indeed, fast ^18^O^16^O^−^ and ^16^O^16^O^−^ peaks are detected, as shown in Fig. 2a,b. In contrast, there is no signal at 36 a.m.u. for all ^18^O_2_ dosing pressures tried, confirming that no molecular ^18^O^18^O^−^ is produced (Fig. 2c). Since sputtered atomic ^18^O^−^ is detected (Fig. 2d), the latter observation signifies that there is no ^18^O_2_ on the Pt surface, whether adsorbed as a whole or formed by recombination of ^18^O atoms. The formation of fast ^18^O^16^O^−^ is easily explained by an ER reaction of H_2_^16^O^+^ abstracting adsorbed ^18^O. The formation of fast ^16^O^16^O^−^ may appear curious at first, as it requires the presence of ^16^O on the surface. But it is readily accounted for by CID of H_2_^16^O^+^ ions^18^, which introduce ^16^O or ^16^OH to the Pt surface. As discussed above, H_2_^16^O^+^ bombardment produces no ^16^O^16^O^−^ signal unless the Pt surface is dosed with molecular oxygen. Water dissociation alone cannot provide enough ^16^O-atom coverage owing to reactions with background hydrogen. Extra ^18^O_2_ exposure reacts off such surface hydrogen, permitting beam-delivered ^16^O to compete with ^18^O atoms for surface sites.

**Figure 3:**
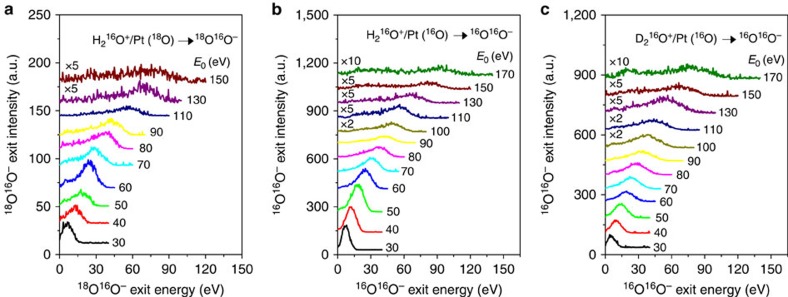
Direct formation of O_2_^−^ in collisions of normal water ions with an oxidized Pt surface. Energy distributions of O_2_^−^ ions produced from (**a**) H_2_O^+^/Pt(^18^O), (**b**) H_2_O^+^/Pt(^16^O) and (**c**) D_2_O^+^/Pt(^16^O). The Pt surface was exposed to ^18^O_2_ or ^16^O_2_ *in situ* at a background pressure of 5 × 10^−8^ torr. Results are shown for multiple incidence energies (*E*_0_) of the corresponding water ions, as indicated. Very weak signal from sputtered O_2_^−^ appears as a second peak for *E*_0_>150 eV.

Energy distributions for ^18^O^16^O^−^ (Fig. 3a), produced from direct abstraction of ^18^O by H_2_^16^O^+^ scattering on Pt(^18^O), are almost identical to those for ^16^O^16^O^−^, produced from H_2_^16^O^+^/Pt(^16^O) (Fig. 3b). In both instances, the product O_2_^−^ peak position varies with H_2_^16^O^+^ incidence energy, confirming further that the observed O_2_^−^ does not originate from sputtering. To avoid confusion, all subsequent references to oxygen—alone or in compounds—will be for ^16^O. Fast O_2_^−^ is also observed from D_2_O^+^/Pt(O) with similar incidence energy dependence (Fig. 3c). The peaks are narrow and well defined, providing accurate measurement of the exit energy. In addition to O_2_^−^, H_2_O^+^/Pt(O) produces scattered H_2_O^+^, O_2_^+^, OH^±^, O^±^, H^+^ and H_2_^+^ (Supplementary Fig. 6). Surviving molecular H_2_O^+^ is only observed for *E*_0_≤100 eV. The O_2_^+^ signal is weak and noisy, indicating that O_2_ mainly exits as O_2_^−^ (neutral O_2_ is not studied). The kinematics of OH^±^ and O^±^ exits can be described by binary collision theory (BCT)^20^, assuming that the collision produces an excited H_2_O, which subsequently dissociates spontaneously (Supplementary Fig. 7).

### O_2_^−^ formation mechanism

**Figure 4:**
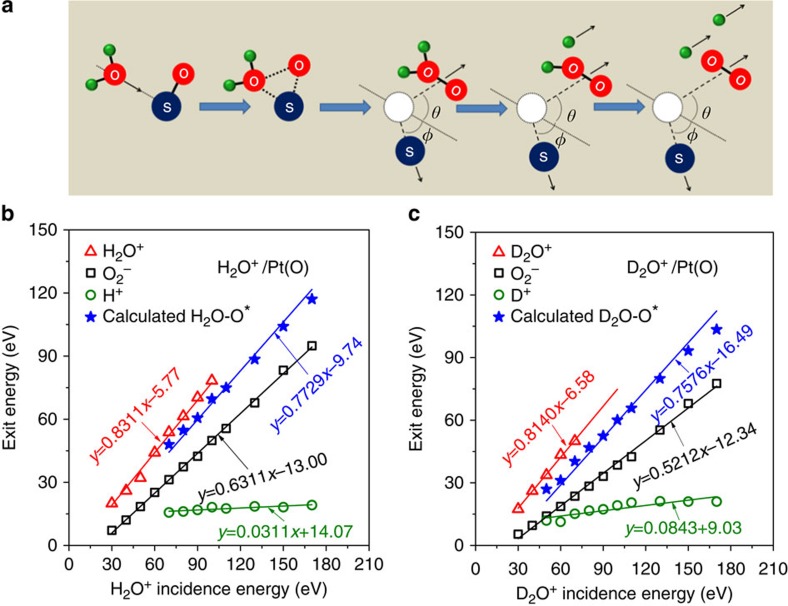
Proposed reaction mechanism and kinematics of direct O_2_ formation from water. (**a**) Schematic depiction of the proposed Eley–Rideal reaction mechanism between energetic water ions and adsorbed O-atoms, producing highly excited oxywater (H_2_O–O* or D_2_O–O*), which undergoes delayed fragmentation to form HO_2_ (DO_2_) as the precursor for O_2_. (**b**) Ion exit energies of H_2_O^+^, O_2_^−^ and H^+^ as a function of H_2_O^+^ incidence energy. The exit energy data of H_2_O–O* were estimated from the measured exit energies of O_2_^−^ and H^+^ (see text). (**c**) Ion exit energies of D_2_O^+^, O_2_^−^ and D^+^ as a function of D_2_O^+^ incidence energy. The exit energy data of D_2_O–O* were estimated (see text). All solid lines in **b**,**c** are linear fittings. The slopes for H_2_O^+^ and D_2_O^+^ are predicted from standard BCT. The slopes for H_2_OO* and D_2_OO* are calculated from a modified BCT model^11^.

We propose that molecular O_2_ forms by means of an ER reaction mechanism driven by energetic H_2_O collisions on oxidized surfaces (Fig. 4a). Following neutralization on approach, a transient state is formed at the distance-of-closest-approach (apsis) between the projectile (H_2_O), the surface atom (S) and the adsorbate (O). Though short-lived, this state promotes a link between projectile and adsorbate. As the projectile rebounds, the transient state disintegrates, producing an energetic molecular product, H_2_O–O*, in an excited state. We conjecture that this product is the elusive oxywater, a structural isomer of hydrogen peroxide and possible intermediate in oxidation reactions initiated by the latter^21^. The H_2_O–O* is unstable^22^ and splits rapidly into a pair of ions, H^+^ and HO_2_^−^, both of which are observed on FeO_*x*_ and SiO_*y*_ (Fig. 1). Weaker HO_2_^−^ signal is also observed for H_2_O^+^/Pt(O) (Supplementary Fig. 6i). The difference in scattered signal intensity from insulating versus metallic surfaces is noteworthy and important for determining HO_2_^−^ abundance in cometary comae (*vide infra*). The corresponding H^+^ fragment possesses surprisingly high kinetic energy, larger than that estimated from mass-weighted energy partitioning of the H_2_O–O* parent. This is remarkable and must be contrasted with the second hydrogen atom, now on HO_2_^−^, which must also be ejected to produce O_2_^−^. This dissociation is promoted by remaining internal energy in the HO_2_^−^ fragment.

The proposed ER reaction mechanism is supported by an analysis of the collisional kinematics. The peak exit energies of H_2_O^+^, O_2_^−^ and H^+^ product ions from H_2_O^+^/Pt(O) are summarized in Fig. 4b, as a function of the H_2_O^+^ incidence energy. Obviously, linear fittings capture the data well. Can the slopes be predicted? First, the kinematics of the H_2_O^+^ exit can be described very well by a slope of 0.8311, which is predicted by BCT when H_2_O^+^ scatters as a whole molecule. The intercepts of such fittings have been correlated to inelastic energy loss, termed ‘inelasticity', associated with the production of a particular excited or ionic state^11^. The H_2_O^+^ inelasticity of ∼5 eV seems small for the production of a highly excited state, so we ascribe this energy loss to a surviving water ion. The kinematics of the O_2_^−^ and H^+^ products cannot be predicted by simple scattering arguments as they are fragments of an excited state, requiring knowledge of how the excitation energy is partitioned when the parent molecule breaks apart.

The critical element of the proposed mechanism is the oxywater transient state, which is not detected in our experiments. If it exists as a parent molecule, its energetics should be reflected in the daughter fragments, H^+^ and HO_2_^−^. A simple summation of their measured kinetic energies may be used to estimate the energy of the H_2_O–O* parent, assuming late fragmentation. Given that HO_2_^−^ signal from scattering on Pt(O) is extremely weak, we must look at the HO_2_^−^ fragments, H and O_2_^−^, to estimate its energy. Since neutral H is not detected, the HO_2_^−^ kinetic energy is estimated from the mass-weighted energy of O_2_^−^ fragment. Finally, the kinetic energy of H_2_O–O* is obtained: *E*(H_2_O–O*)=33/32·*E*(O_2_^−^)+*E*(H^+^). This simple formula produces a number of points (Fig. 4b), which can be fitted relatively well by a straight line with a slope of 0.7729, corresponding to the kinematic factor predicted from BCT for H_2_O–O* formation by an ER reaction^11^.

The estimation method for the exit energy of the transient state is validated with data for D_2_O^+^/Pt(O). The peak exit energies of the D_2_O^+^, O_2_^−^ and D^+^ product ions are summarized in Fig. 4c, as a function of the D_2_O^+^ incidence energy. Again, the kinematics of the D_2_O^+^ exit is described well by the predicted kinematic factor of 0.8140, when D_2_O^+^ scatters intact. The formula for calculating the D_2_O–O* exit energy now becomes: *E*(D_2_O–O*)=34/32·*E*(O_2_^−^)+*E*(D^+^). The calculated points, shown in Fig. 3c, can be fitted by a straight line with a predicted^11^ slope of 0.7576, though not as well as before.

The slopes of the data for O_2_^−^, H^+^ (Fig. 4b) and O_2_^−^, and D^+^ (Fig. 4c) cannot yet be predicted. Unconstraint, two-parameter linear fitting captures the data very well, suggesting a simple excited-energy partitioning mechanism between the oxywater molecule fragments. Comparing inelasticities, it appears that about the same energy is consumed to form O_2_^−^ from H_2_O^+^ versus D_2_O^+^. The H^+^ and D^+^ fittings (currently not understood) show positive inelasticity, implying an energy gain. Most of the internal energy of the oxywater transient state is likely converted into kinetic energy for the lighter fragments^23^.

Similar ER reaction mechanisms yielding O_2_^−^ product also occur for OH^+^, OD^+^ and O^+^ bombardment of Pt(O) (Supplementary Fig. 8). The O_2_^−^ exit energy data versus incidence energy for all incident ions can be described very well by BCT, confirming the validity of the kinematic analysis (Supplementary Fig. 9). The OH^+^ and O^+^ species are important because the ‘accelerated water ions,' found in the 67P coma, include ions with molecular mass between 16 and 19 a.m.u. (ref. 12). Some of these H_2_O^+^ dissociation fragments are re-introduced into the coma, where the solar wind may pick them up and recycle them back to the comet surface, thus increasing O_2_ production.

## Discussion

We have uncovered high-energy reaction channels for dynamic production of negative ions from collisions of energetic water ions with oxidized surfaces. The latter surfaces include: SiO_*x*_, FeO_*y*_, Pt(O), NiO_*z*_, Pd(O), Au(O) and TiO_*w*_ (see also Supplementary Figs 10–12). Such interactions are applicable to plasmas and astrophysical environments whenever H_2_O^+^ ions are encountered with kinetic energies between 50 and 300 eV. We propose that the scattering interactions occur in cometary comae during periods of activity, where they produce energetic negative ions, including: O^−^, OH^−^, O_2_^−^ and HO_2_^−^. The latter two ions, in particular, are produced by a novel ER reaction, and contribute to the O_2_ abundance in the coma after photodetachment^24^,^25^. The lifetime of O_2_^−^ against photo-detachment is 2.6 s (at 1 a.u.)^25^, which suggests that O_2_^−^ should be able to reach Rosetta. Negative ions should be present in the coma of 67P but they have yet to be reported, baring H^−^ (ref. 26). Thus, our work actually predicts the existence of O_2_^−^ and HO_2_^−^ in the coma at distances sufficiently close to the nucleus to avoid photodetachment. Negative ions of cometary origin have been detected in the coma of comet 1P/Halley, though without sufficient mass resolution to distinguish individual ions^25^. Three broad peaks were observed, which were denoted as the 17-, 30- and 100-a.m.u. peaks. Chaizy *et al*.^24^ argued that the first peak included O^−^ and OH^−^, while the second peak comprised CN^−^. Furthermore, these authors considered several negative ion production mechanisms and found them inadequate to explain the signal intensity observed in the Halley coma. We propose that energetic water ion scattering off of the nucleus surface or dust grains in the coma of 1P/Halley could populate both the 17- and 30-a.m.u. peaks, and that the latter peak must have also included O_2_^−^ and HO_2_^−^.

The ER reaction mechanism is consistent with most reported^6^ cometary O_2_ observations and trends and must be an important contributor to its abundance. All necessary conditions for ER reactions on comet 67P are met: (i) water ions with the correct hyperthermal energies exist in the coma, and (ii) they impact the nucleus or dust grain surfaces, which (iii) contain oxidized materials. The mechanism explains well the strong correlation of O_2_ to water abundance in the coma, and also the O_2_ signal increase closer to the nucleus—scattering makes the surface appear as a point source of O_2_, thus justifying the observed 1/*r*^2^ dependence, where *r*=cometocentric distance. The connection to solar wind may account for the relative invariance in the O_2_/H_2_O ratio with heliocentric distance. That is, as the comet approaches the Sun, more subsurface sublimation leads to more water molecules in the coma; the solar wind also strengthens, increasing ionization and water ion flux to the surface, ultimately producing more O_2_. The surface reaction is independent of the nucleus surface temperature or comet illumination conditions. The ER mechanism goes beyond these trends to explain the presence of significant amounts of HO_2_ and the absence of O_3_, which have baffled Bieler *et al*.^6^ The findings are generic to comets irrespective of their origin in the early Solar System^27^.

The primordial origin of cometary O_2_ requires first a mechanism for O_2_ formation. Water ice radiolysis by galactic cosmic rays during primordial times has been suggested^6^ as that mechanism, despite evidence for very low O_2_ abundance in protostellar envelopes^28^. Radiolysis is known to produce the chemically related species O_3_, H_2_O_2_ and HO_2_ (refs 29, 30, 31). The former two molecules are stable and should have also been incorporated into the comet at the same time as O_2_. However, no O_3_ has been detected in the 67P coma, a concern identified by Bieler *et al*.^6^ that also applies to other efforts at explaining the primordial origin^32^,^33^,^34^. On the other hand, H_2_O_2_ and HO_2_ have been detected and their gaseous abundance ratios were reported for the 67P coma: H_2_O_2_/O_2_=0.6 × 10^−3^ and HO_2_/O_2_=1.9 × 10^−3^. These were compared with the abundance ratios measured in the *ρ* Oph A dense core^29^, where O_2_ has also been detected and is likely to originate from radiolysis: H_2_O_2_/O_2_≈HO_2_/O_2_≈0.6 × 10^−3^. The H_2_O_2_ abundance relative to O_2_ is clearly a perfect match but the value for the HO_2_/O_2_ ratio is 3 × larger in the coma of 67P. This difference suggests that HO_2_ is formed at higher rates than it can be destroyed, thus accumulating in the coma. It is likely that HO_2_ forms by a mechanism different from or perhaps in addition to that operating under interstellar conditions. Apart from the mechanism discussed in this communication, there is actually another reaction mechanism enabled by the presence of O_2_ in the coma, which involves a different ER reaction^35^. Like H_2_O^+^, photo-ionized O_2_^+^ can be picked up by solar wind and accelerated back to the comet, where it can abstract atomic H from cometary materials to form HO_2_.

There have been other attempts to justify the primordial O_2_ formation and its survival for 4.6 billion years. Mousis *et al*.^32^ considered the radiolysis of icy grains in the low-density proto-solar nebula, which may produce large amounts of O_2_ though ‘its incorporation as crystalline ice is highly implausible'. These authors discussed two extreme O_2_ production scenarios for dense and early proto-solar nebula, which require very large galactic cosmic ray fluxes and O_2_ trapping in clathrates. Taquet *et al*.^33^ used sophisticated astrochemical models to compare various primordial O_2_ formation mechanisms, proposing oxygen atom recombination at the surface of interstellar ices as a possibility, albeit under ‘warmer and denser conditions than usually expected in dark clouds'. Finally, Dulieu *at al.*^34^ proposed that O_2_ forms *in situ* during the evaporation of water ice via a dismutation reaction of co-evaporating H_2_O_2_. This mechanism requires the incorporation of primordial H_2_O_2_ in large amounts into the nucleus and its complete conversion into O_2_ to be consistent with the low levels of H_2_O_2_ in the coma. All these mechanisms appear to be in conflict with the relative abundances of the related species O_3_, H_2_O_2_ and HO_2_.

In contrast, the ER reaction mechanism comprises cometary ions and minerals actually found on 67P. It produces O_2_ *in situ* through the putative oxywater state, which dissociates spontaneously into HO_2_^−^ as an intermediate on its way to O_2_^−^ or HO_2_ following photodetachment. That is, HO_2_ is co-produced by this new reaction and should add to any amounts formed by other mechanisms. It is reasonable then to expect that the HO_2_/O_2_ ratio should be larger than interstellar values^30^. The remaining factor, H_2_O_2_, can form collisionally in the extended coma by hydrogen atom transfer from scattered HO_2_^−^ or HO_2_ to ambient H_2_O. Finally, no O_3_ is produced during the ER reaction, consistent with observations.

The crux of any proposed O_2_ production mechanism is whether it can explain the observed O_2_ abundance in the 67P coma. The O_2_^−^ production rate by the ER reaction is proportional to the accelerated water ion flux, which has been measured to be 3 × 10^9^–3 × 10^11^ m^−2^ s^−1^ at 2 a.u. with the caveat that it may be underestimated by at least two orders of magnitude^13^,^36^. The proportionality constant (O_2_ yield) cannot be estimated at this time. Regardless, the reported flux is too low to make ‘accelerated' water ions entirely responsible for the reported O_2_ abundance. However, it is noteworthy that there are other water-derived ions present in the coma, which can participate in similar ER reactions on the nucleus surface. For example, the extended coma also contains abundant H_3_O^+^, OH^+^ and O^+^ (ref. 36), all of which can be picked up by the solar wind and accelerated to energies sufficient to drive ER reactions (Supplementary Figs 8 and 9). Unfortunately, the flux and energy distributions of these additional ions have not been reported—H_3_O^+^, in particular, may be a serious contributor as its density in the inner coma varies considerably and can reach 100 times the density of H_2_O^+^ (ref. 37). Furthermore, the ‘cold' water ions^5^ have two orders of magnitude larger flux and possess kinetic energies up to 50 eV but move away from the comet, so they can produce O_2_ only in collisions with dust grains. Without some knowledge of these additional species flux and energy distributions and of the dust grain density in the coma, we cannot quantify the magnitude of the ER reaction contributions to the cometary O_2_.

Nevertheless, we note that a unique feature of the ER reaction mechanism is its ability to produce energetic O_2_^−^ anions, moving away from the nucleus towards the orbiting Rosetta spacecraft with kinetic energy between 10 and 50 eV. The Rosetta double focus mass spectrometer (ROSINA/DFMS) entrance slit plate is biased in gas mode to reject ambient ions^38^. Positive bias will attract and accelerate O_2_^−^ into the ionizer box. Depending on cometocentric distance, some or all of the O_2_^−^ anions in transit to Rosetta will undergo photodetachment^24^, producing neutral O_2_ molecules, which retain their kinetic energy and will enter the ionizer regardless of bias. Energetic collisions of hyperthermal O_2_^−^ ions or neutralized O_2_ molecules with the gold-coated internal surfaces of the ROSINA/DFMS ionizer will produce O_2_^+^ ions by surface re-ionization^39^ (Supplementary Fig. 11). It is worth pondering how O_2_^+^ formed inside the ROSINA/DFMS by a mechanism other than electron impact ionization will contribute to the detected O_2_^+^ signal^40^.

In conclusion, energetic water ions in cometary comae, produced and accelerated by solar wind, can drive scattering interactions on cometary surfaces that alter the relative speciation in the coma. CID of water ions on the comet can generate negative ions (O^−^ and OH^−^). Abstraction of chemisorbed oxygen from oxidized surfaces by water ions can also produce dynamically O_2_^−^ and HO_2_^−^, by means of a previously unknown ER reaction. Kinematic analysis of the ER reaction products provides indirect evidence for the elusive oxywater state as a reaction intermediate, which may form during the hard collision, then dissociate promptly on the rebound from the surface. When this reaction occurs in comets, it can populate the coma with energetic O_2_^−^ anions, which are converted readily to molecular O_2_ by photo-detachment. This abiotic way to produce molecular O_2_ informs our understanding of cometary chemistry and could be important in other astrophysical environments.

## Methods

### Materials

Polycrystalline Pt and Fe foils (4 N purity, ESPI) were used as received or sputter-cleaned *in situ* as needed. Doped Si wafers (n-type) covered with native silicon oxide (thickness 2–3 nm) were degreased but otherwise left untreated. Research grade ^16^O_2_ and ^18^O_2_ gases (5 N) were used for the dosing experiments.

### Scattering apparatus operation

Laboratory experiments were carried out in an ultra-high vacuum ion scattering system connected to an ion beam line, as described in detail elsewhere^41^,^42^. Positive ions were extracted from an inductively coupled plasma of H_2_O or D_2_O in Argon carrier gas, operated at 5 mtorr and 500 W of radio-frequency power supplied at 13.56 MHz. The ions were launched into the ion beam line at −15 KV and were magnetically mass-filtered to produce isotopically pure beams of O^+^, OH^+^, OD^+^, H_2_O^+^ and D_2_O^+^, with fluxes between 2 and 5 μA. Ion energy was tuned by adjusting the plasma potential with respect to ground using external bias. The energy width of all incident beams was constant at ∼5 eV (full width at half maximum), determined by the electron temperature in the plasma. The ion beams were then decelerated and delivered to grounded target surfaces, held at room temperature. The beam waist on the sample was ∼3 mm. Gas dosing of the target surfaces was accomplished using a tube situated ∼2 cm from the surface. O-atom coverage was adjusted (but not measured) by changing the background O_2_ pressure using a leak valve. The ions impinged on the surface at 45° angle of incidence and the scattered products were detected at 45° angle of exit in the scattering plane. Though some incident ions survive the collision as charged species, most are typically neutralized efficiently on the incoming trajectory via charge exchange with the surface^43^. The violent collision with the metal surface causes hyperthermal surface ionization^39^, a process that allows some of the scattered products to exit the surface as positive or negative ions without resorting to electron-impact or photo-ionization schemes. In fact, studies of scattering at high energies would not be possible without surface ionization, as most conventional electron-impact ionizers are very inefficient at high product exit velocities. It is for this reason that no neutral products could be resolved in continuous wave experiments; their presence has been verified in calibration experiments by pulsing the beam and using lock-in detection schemes. All charged scattered products were chemically identified with a high-resolution mass spectrometer (Extrel QPS) and their translational energies were measured using a calibrated 90^o^-sector energy analyser. All signals reported were normalized to the corresponding beam current.

### Cometary surface analogues

Thin oxides are used as analogues of cometary materials in order to avoid localized surface charging, which occurs during beam exposure at high ion flux. Surface charging interferes with the measurement of ion exit energies. When using native silicon or iron oxide (∼2–3 nm), electron tunnelling to the underlying conductive substrate allows removal of the surface charge. The presence of solar wind in the cometary environment mitigates this problem, by providing electrons to the exposed cometary materials (silicates, quartz and so on) to neutralize this charge. The choice of the oxides is obvious: (1) oxygen atoms on the silicon oxide surface are bonded to Si in a similar fashion to surface oxygen atoms in silicates, and (2) elemental Fe has been found on 67P, and given the oxidizing conditions in the coma, oxidized Fe surfaces are to be expected. Furthermore, the charging effect has been verified by scattering on titanium oxide, which is a semiconductor and has mild conductivity, but exhibits the same scattering behaviour as silicon oxide (Supplementary Fig. 12).

### Data availability

All relevant data supporting the findings of this study are available from the corresponding author upon request.

## Additional information

**How to cite this article:** Yao, Y. & Giapis, K. P. Dynamic molecular oxygen production in cometary comae. *Nat. Commun.* **8,** 15298 doi: 10.1038/ncomms15298 (2017).

**Publisher's note:** Springer Nature remains neutral with regard to jurisdictional claims in published maps and institutional affiliations.

## Supplementary Material

Supplementary Information Supplementary Figures and Supplementary Table

Peer Review File
