# Supplementary material for: Dynamic molecular oxygen production in cometary comae
Source: Nat Commun. 2017 May 8;8:15298. doi: 10.1038/ncomms15298 (PMC5424151; doi:10.1038/ncomms15298)
Supplement: Supplementary Information — Supplementary Figures and Supplementary Table [file ncomms15298-s1.pdf]

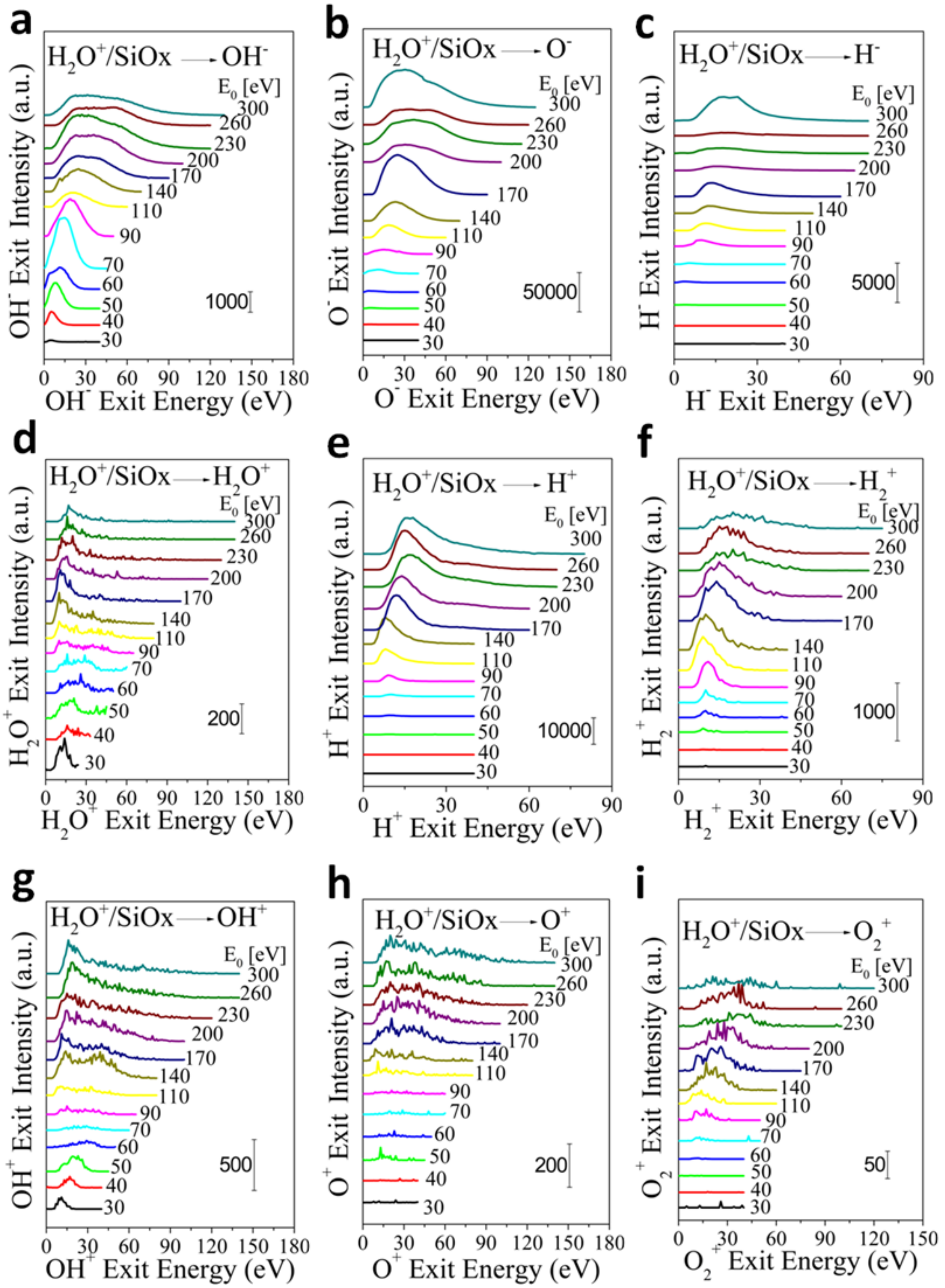

Supplementary Figure 1. Negative and positive ion exits from H_2O^+ scattering on native Si oxide. Energy distribution of ion products from H_2O^+/SiOx: (**a**) OH^-, (**b**) O^-, (**c**) H^-, (**d**) H_2O^+, (**e**) H^+, (**f**) H_2^+, (**g**) OH^+, (**h**) O^+ and (**i**) O_2^+. Multiple curves on each panel correspond to scattering experiments at different incident energies, as indicated.

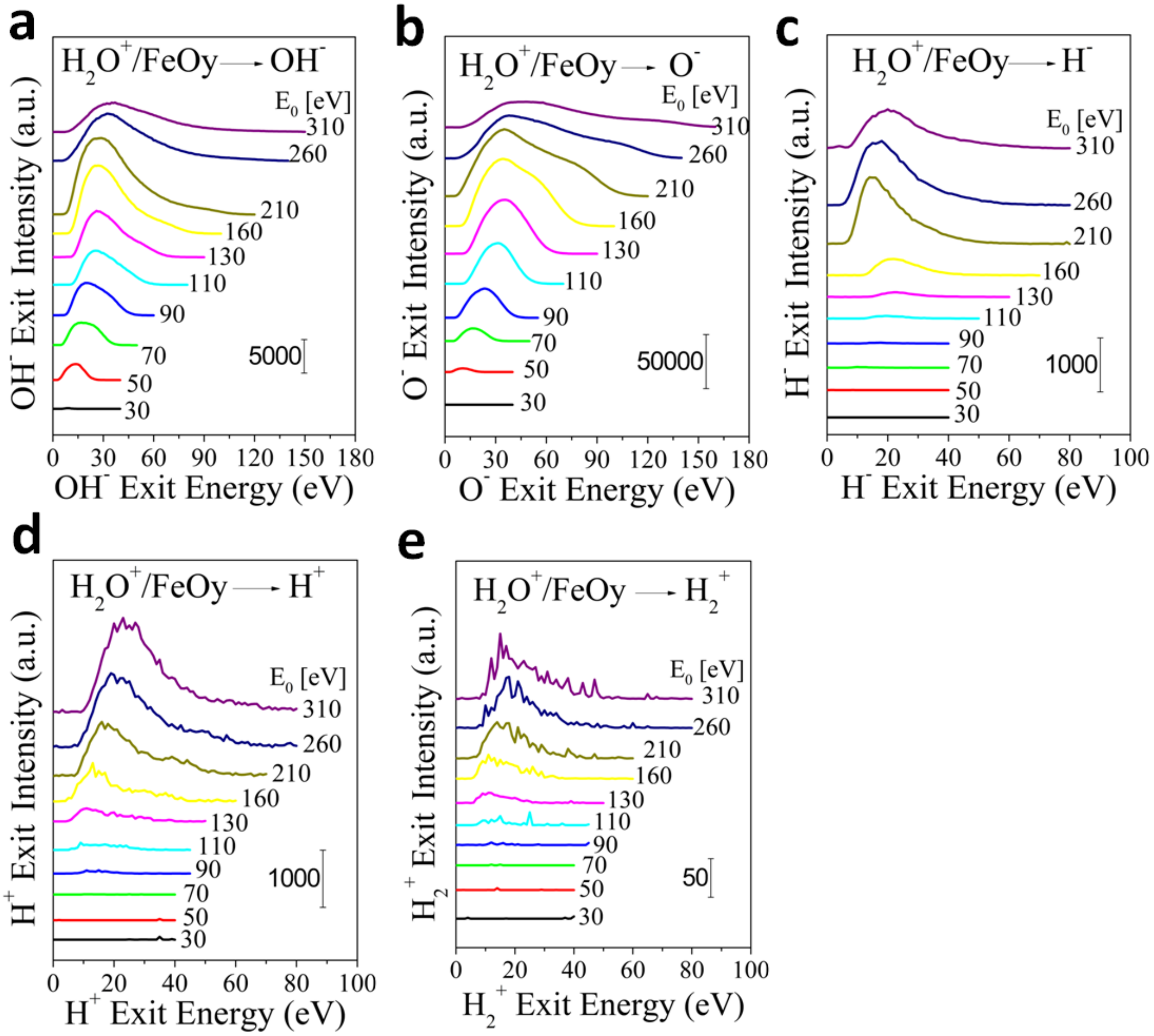

Supplementary Figure 2. Negative and positive ion exits from H_2O^+ scattering on native Fe oxide. Energy distribution of ion products from H_2O^+/FeO_y: (**a**) OH^-, (**b**) O^-, (**c**) H^-, (**d**) H^+, and (**e**) H_2^+. Multiple curves on each panel correspond to scattering experiments at different incident energies, as indicated. Except for H^+ and H_2^+, all product ions off of FeO_y are negatively charged.

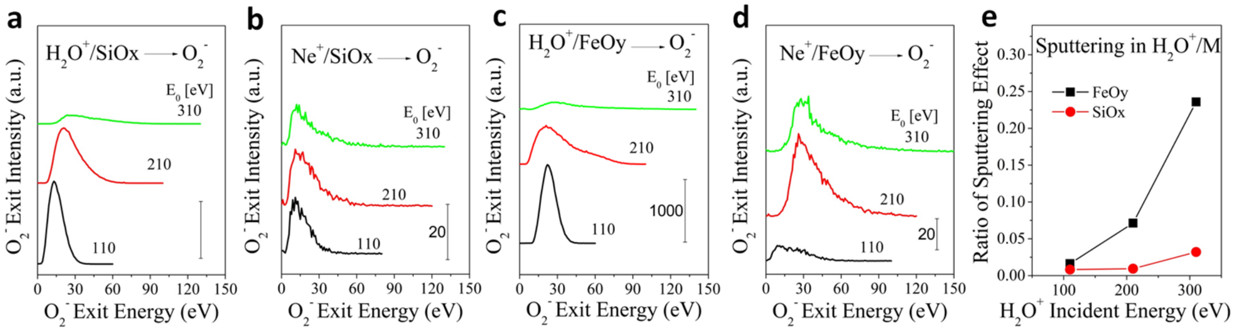

Supplementary Figure 3. Reactive versus non-reactive (sputtering) contributions to O_2^{-} formation. Energy distributions of O_2^{-} produced at E_0=310 eV from: (a) H_2O^+/SiOx, (b) Ne^+/SiOx, (c) H_2O^+/FeOy, and (d) Ne^+/FeOy. Results are compared for three incident energies as indicated. All distributions are normalized to the corresponding ion beam current on the sample. (e) Ratios of the integrated O_2^{-} signal intensities, produced from Ne^+ vs. H_2O^+ scattering on the same surface, as a function of the incident energy. Sputtering contributions increase with energy as expected.

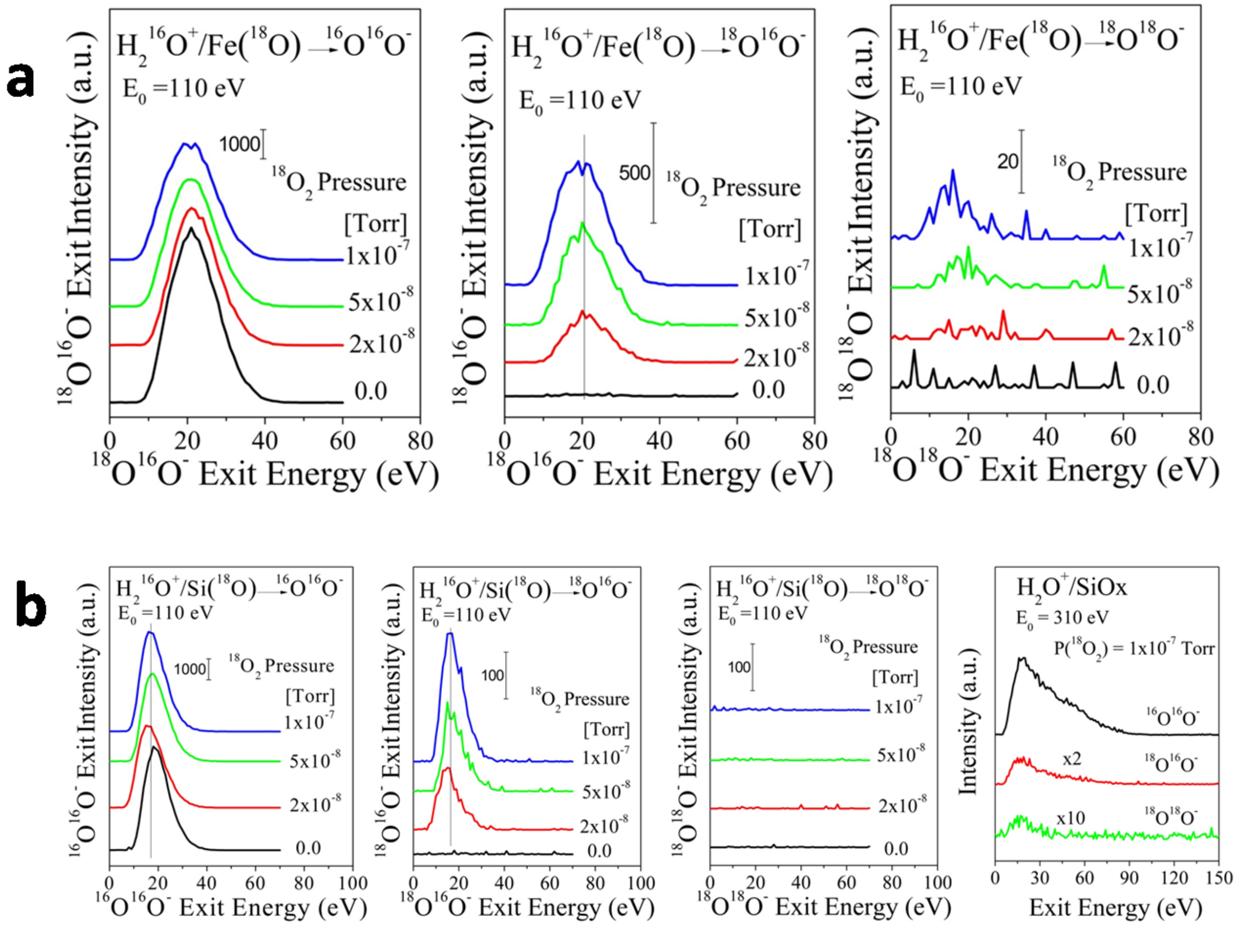

Supplementary Figure 4. Isotopic O_2^- products from normal water ion ($H_2{}^{16}O^+$) scattering on ^{18}O-covered FeO_y and SiO_x surfaces. When the surface is partially covered with ^{18}O atoms, both $^{16}O^{16}O^-$ and $^{18}O^{16}O^-$ can be produced by ER reactions between $H_2{}^{16}O^+$ and surface adsorbed ^{16}O or ^{18}O atoms. But $^{18}O^{18}O^-$ can only be produced by surface sputtering. The sputtering signal intensity is then directly related to the surface population of ^{18}O (coverage=θ), which competes with ^{16}O (coverage=1-θ) for sites, assuming zero empty sites. From statistics, the sputtering peak intensity should follow $I(^{18}O^{18}O^-): I(^{18}O^{16}O^-): I(^{16}O^{16}O^-) = \theta^2: 2\theta(1-\theta): (1-\theta)^2$. Thus, by measuring the $^{18}O^{18}O^-$ sputtering peak intensity, the sputtering contributions to $^{18}O^{16}O^-$ and $^{16}O^{16}O^-$ can be calculated. In (**a**) H_2O^+/FeOy and (**b**) H_2O^+/SiOx, the surface ^{16}O

was partially replaced by ^{18}O by in situ exposure to $^{18}O_2$. When the direct ER reaction becomes dominant, the relative population of ^{18}O (θ) can be estimated from θ:(1-θ)= I($^{18}O^{16}O^-$): I($^{16}O^{16}O^-$). Performing this analysis for data collected at E_0=110 eV, the sputtering contribution to O_2^- from H_2O^+/FeOy is estimated to be 15% (**Supplementary Table 1a**). There is no $^{18}O^{18}O^-$ detected for H_2O^+/SiOx at E_0 =110 eV under $^{18}O_2$ dosing, that is, sputtering does not contribute significantly to O_2^- formation on SiOx at this energy. However, the situation is very different at higher energies when the ER reaction dies out. For example, a $^{18}O^{18}O^-$ sputtering peak is observed at E_0=310 eV. Based on the above analysis, the sputtering contribution to O_2^- at this energy is estimated to be 77% (**Supplementary Table 1b**), consistent with the experiment: the direct O_2^- formation channel dies out at higher incident energies. Since physical sputtering dominates the O_2^- production at E_0=310 eV, the calculated sputtering contribution of 77% is underestimated.

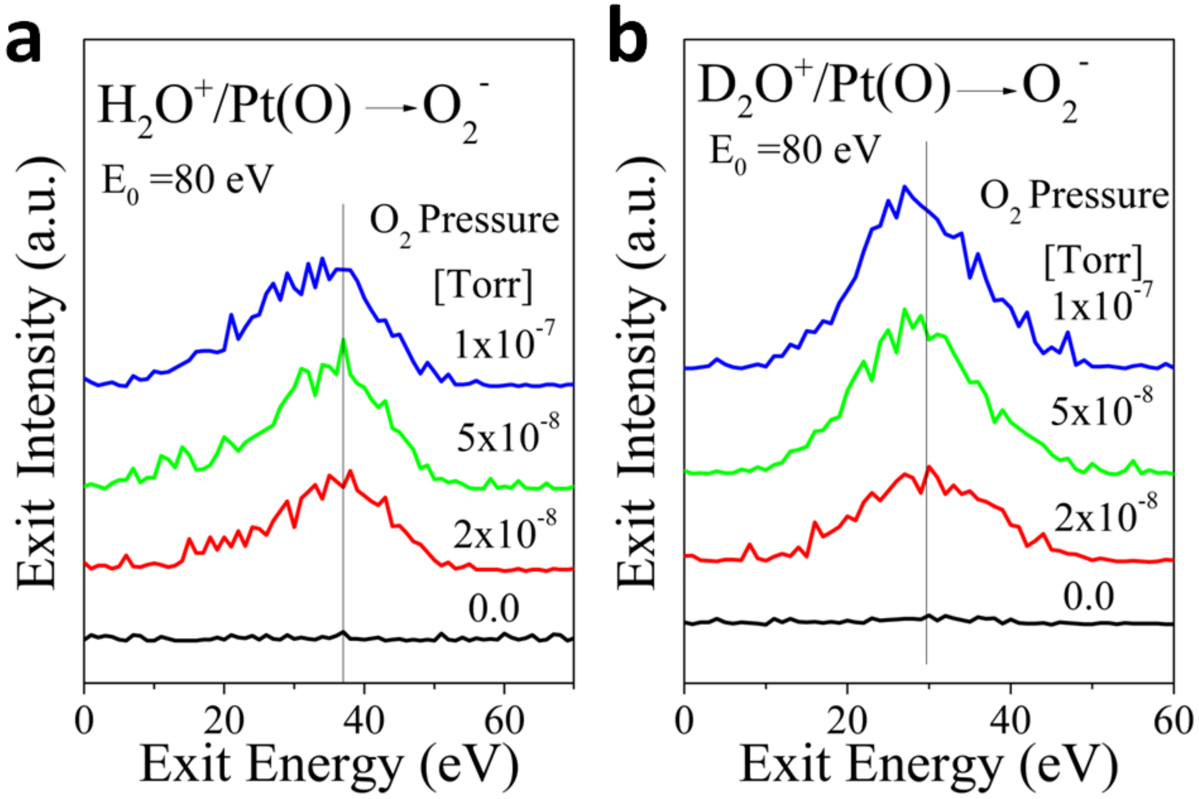

Supplementary Figure 5. Oxygen exposure effect on O_2^- formation from normal versus heavy water ions. Energy distributions of O_2^- ion exits from (**a**) H_2O^+ and (**b**) D_2O^+ scattering on Pt at various O_2 exposure pressures, as indicated. The incident energy is fixed at E_0=80eV.

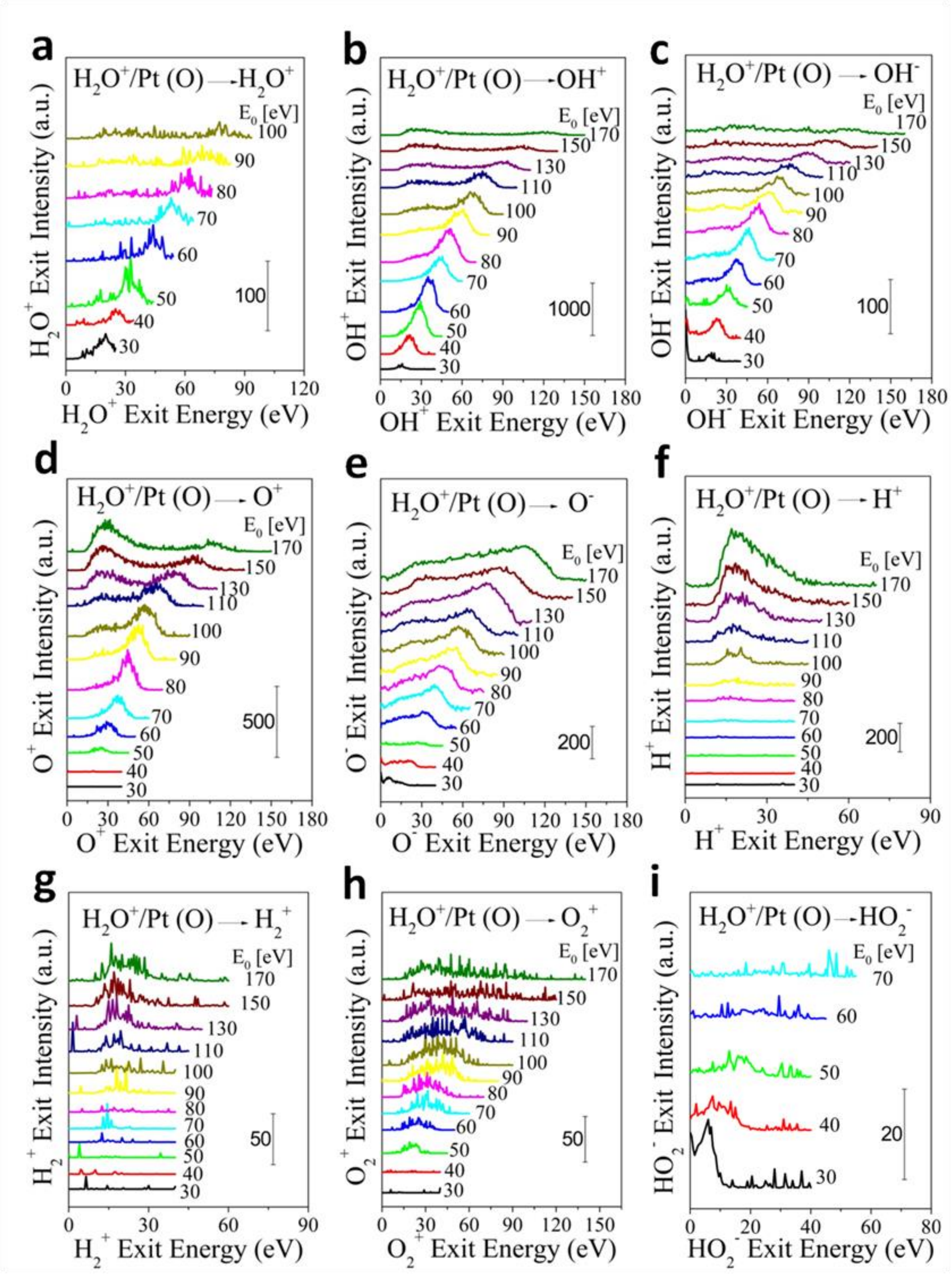

Supplementary Figure 6. Additional ion products from H_2O^+ scattering on Pt covered with O atoms. Energy distributions of ion exits from H_2O^+/Pt(O): (**a**) H_2O^+, (**b**) OH^+, (**c**) OH^-, (**d**) O^+, (**e**) O^-, (**f**) H^+, (**g**) H_2^+ , (**h**) O_2^+ and (**i**) HO_2^- at various H_2O^+ incident energies.

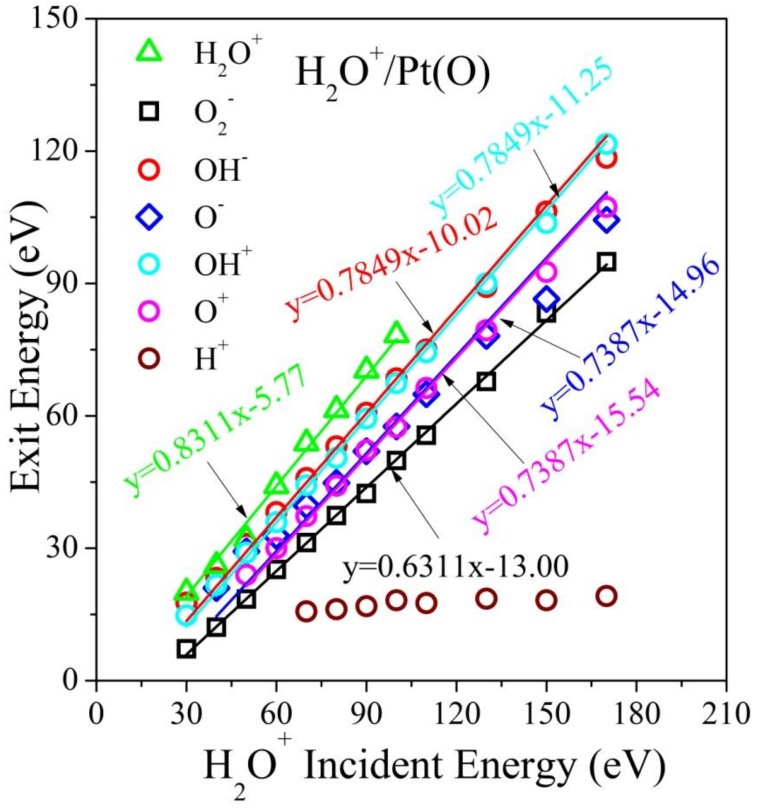

Supplementary Figure 7. Kinematics of ion exits from H_2O^+ scattering on Pt covered with O atoms. The solid lines are linear fittings. The slope for H_2O^+ is calculated from binary collision theory by assuming the H_2O scatters as a whole projectile (scattering angle =90°). The slopes for OH^+, OH^-, O^+ and O^- are mass ratios of the kinematic factor of scattered H_2O^+. The kinematic data of O_2^- are linearly fitted without constraints (two-parameters).

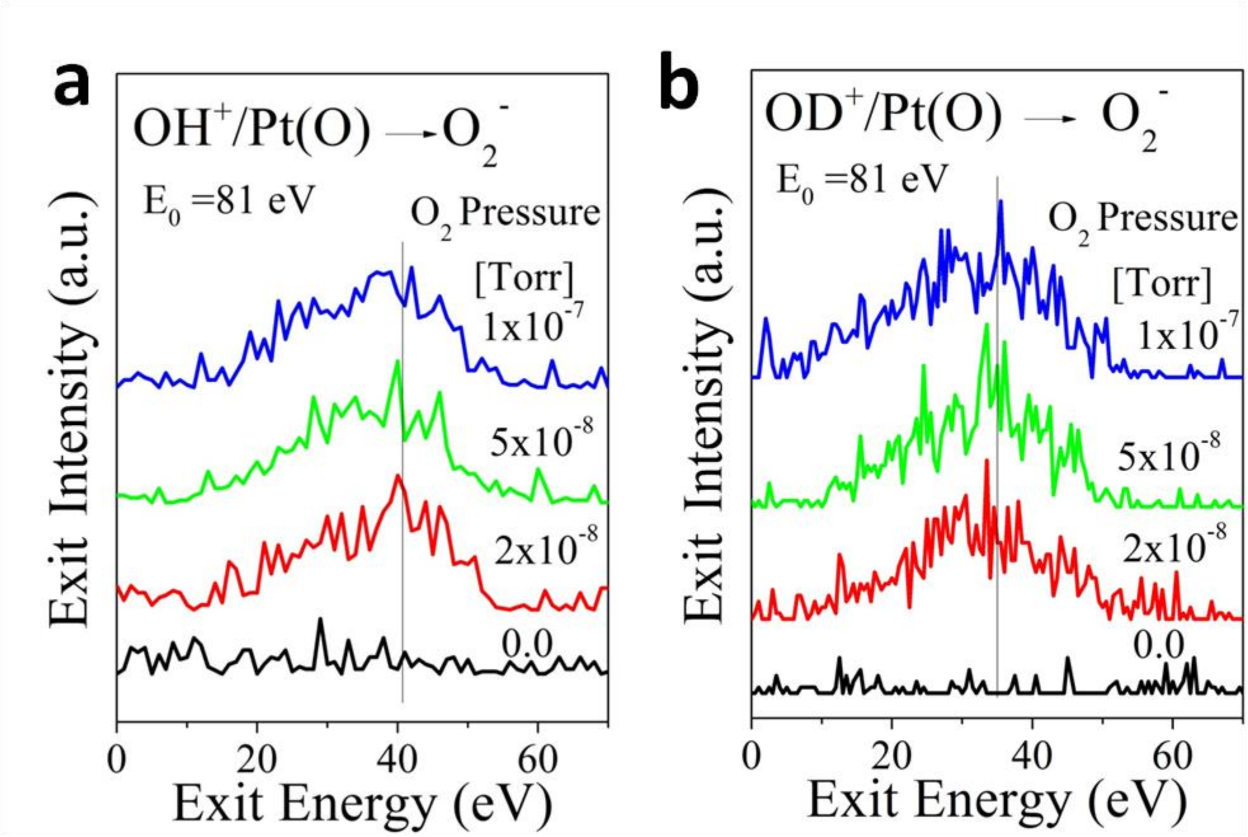

Supplementary Figure 8. O_2^- production from OH^+ and OD^+ scattering on Pt(O). Energy distribution of O_2^- ion exits from: (**a**) OH^+ and (**b**) OD^+ scattering on Pt at various O_2 exposure pressures, as indicated. The incident energy is fixed at E_0=81eV.

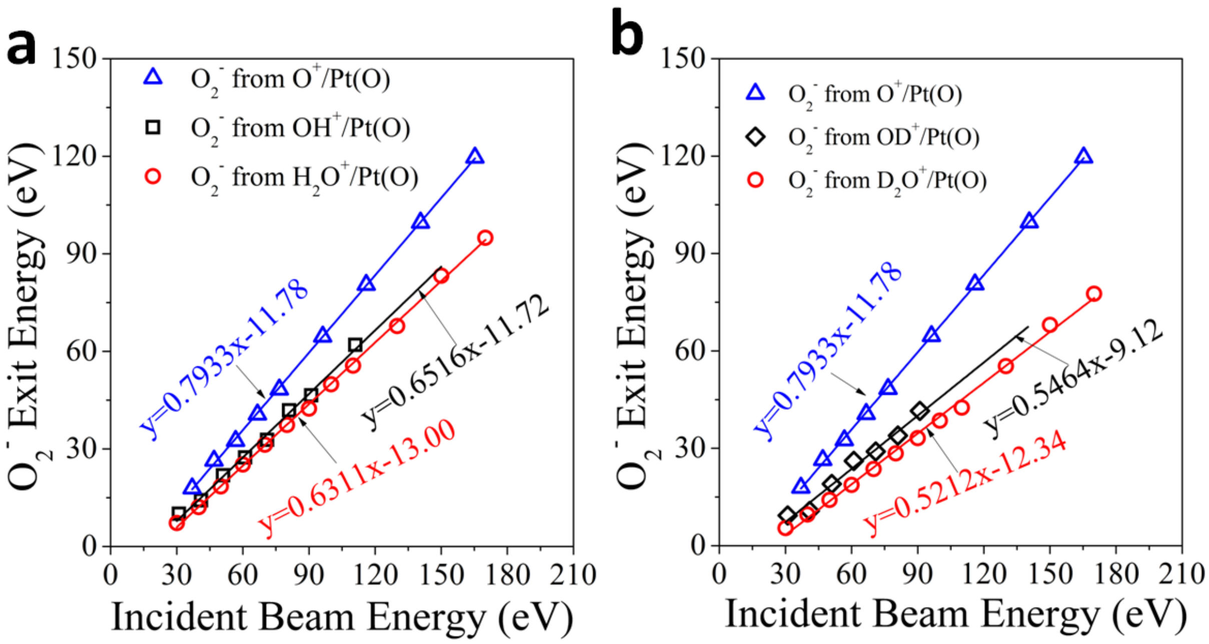

Supplementary Figure 9. Comparison of the kinematics of O_2^- formation from water ions and fragments thereof on Pt(O). (a) Ion exit energies of O_2^- from O^+, OH^+ and H_2O^+ scattering on Pt(O) as a function of corresponding ion incident energy. **(b)** Ion exit energies of O_2^- from O^+, OD^+ and D_2O^+ scattering on Pt(O) as a function of corresponding ion incident energy. The solid lines are linear fitting. The slope for O_2^- from O^+/Pt(O) is calculated using a modification to the Binary Collision Theory (BCT) explained in ref. 11.

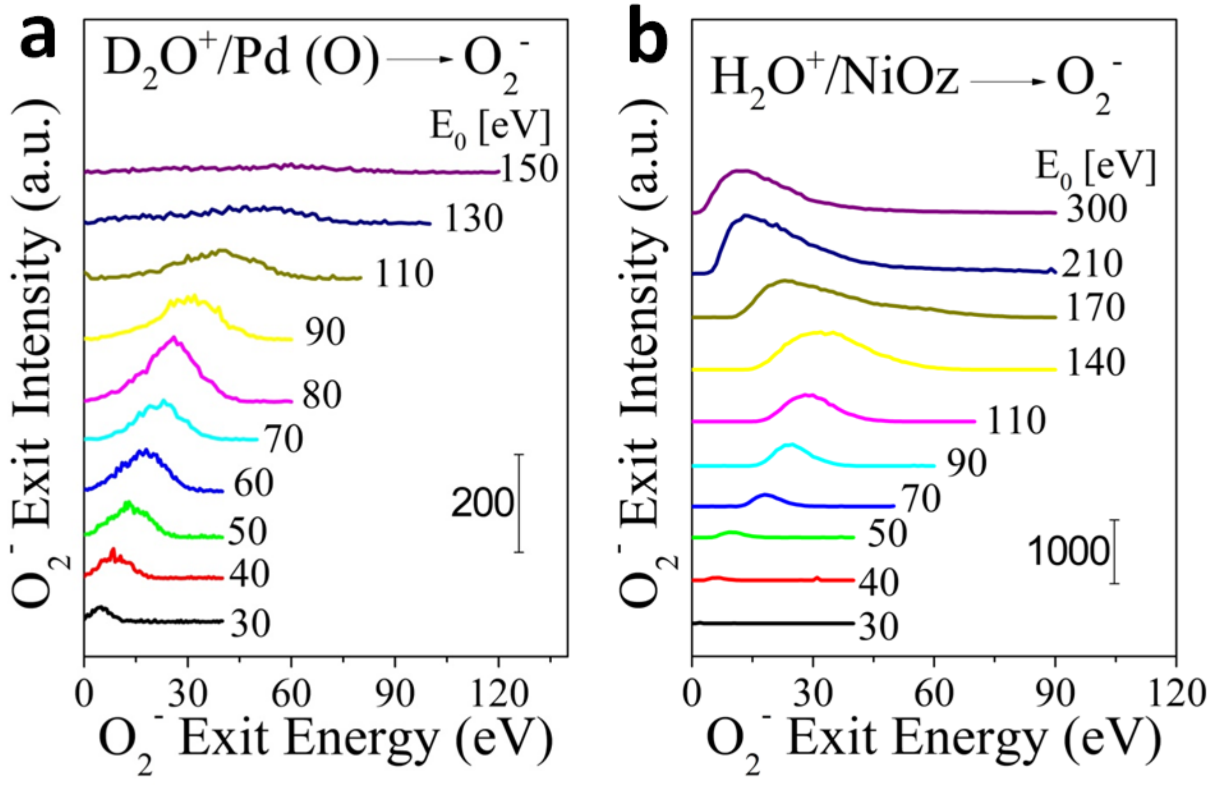

Supplementary Figure 10. O_2^- formation in water ion scattering on Pd(O) and native Ni oxide. (a) Energy distributions of O_2^- from D_2O^+ scattering on Pd(O). The surface O atoms were produced by in situ O_2 exposure at 5×10^{-8} Torr. The O_2^- peak position shifts monotonically to higher energy with increasing E_0. **b,** Energy distributions of O_2^- from H_2O^+ scattering on native Ni oxide (NiOz). The O_2^- peak position first shifts to higher energy with E_0 up to 140 eV, then decreases. The direct ER reaction channel between H_2O^+ and surface O dies out at high $E_{0,}$ and physical sputtering becomes more pronounced.

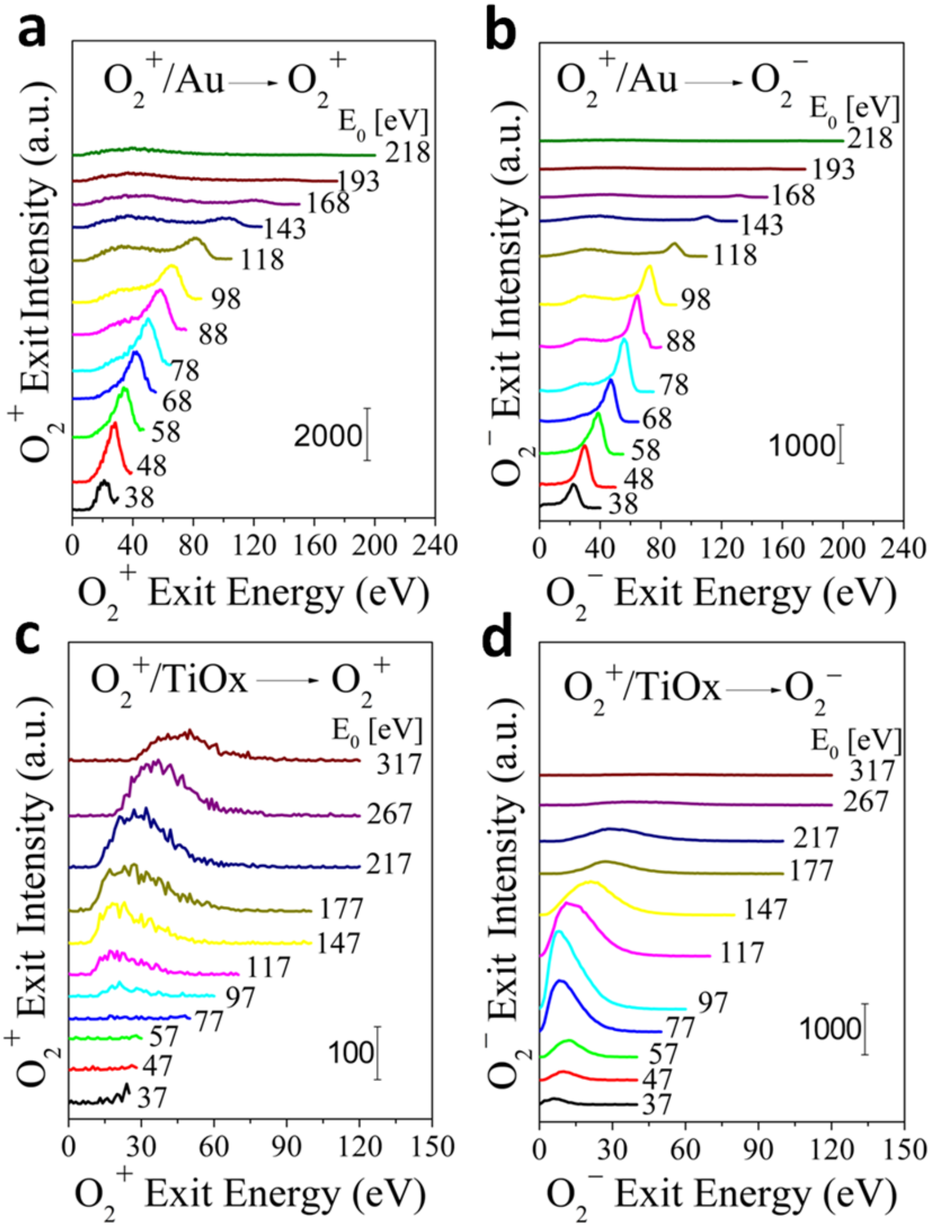

Supplementary Figure 11. O_2^+ scattering on Au and on native titanium oxide. Energy distributions of (**a**) O_2^+, (**b**) O_2^- from O_2^+ scattering on Au, (**c**) O_2^+ and (**d**) O_2^- from O_2^+ scattering on native Ti oxide. Results are shown for positive ion collisions, though negative ion collisions are discussed in the text. Both ion polarities become neutralized on approach to the surface very efficiently (~98%) by charge transfer mechanisms. Re-ionization typically occurs on the rebound, either via auto-ionization of an excited state formed during the collision, or by resonant charge transfer. O_2^+ production from O_2^+/Au, shown in (**a**), is very efficient and occurs at very low incidence energies (O_2^-/Au should be very similar). O_2^+ is also produced on TiOx.

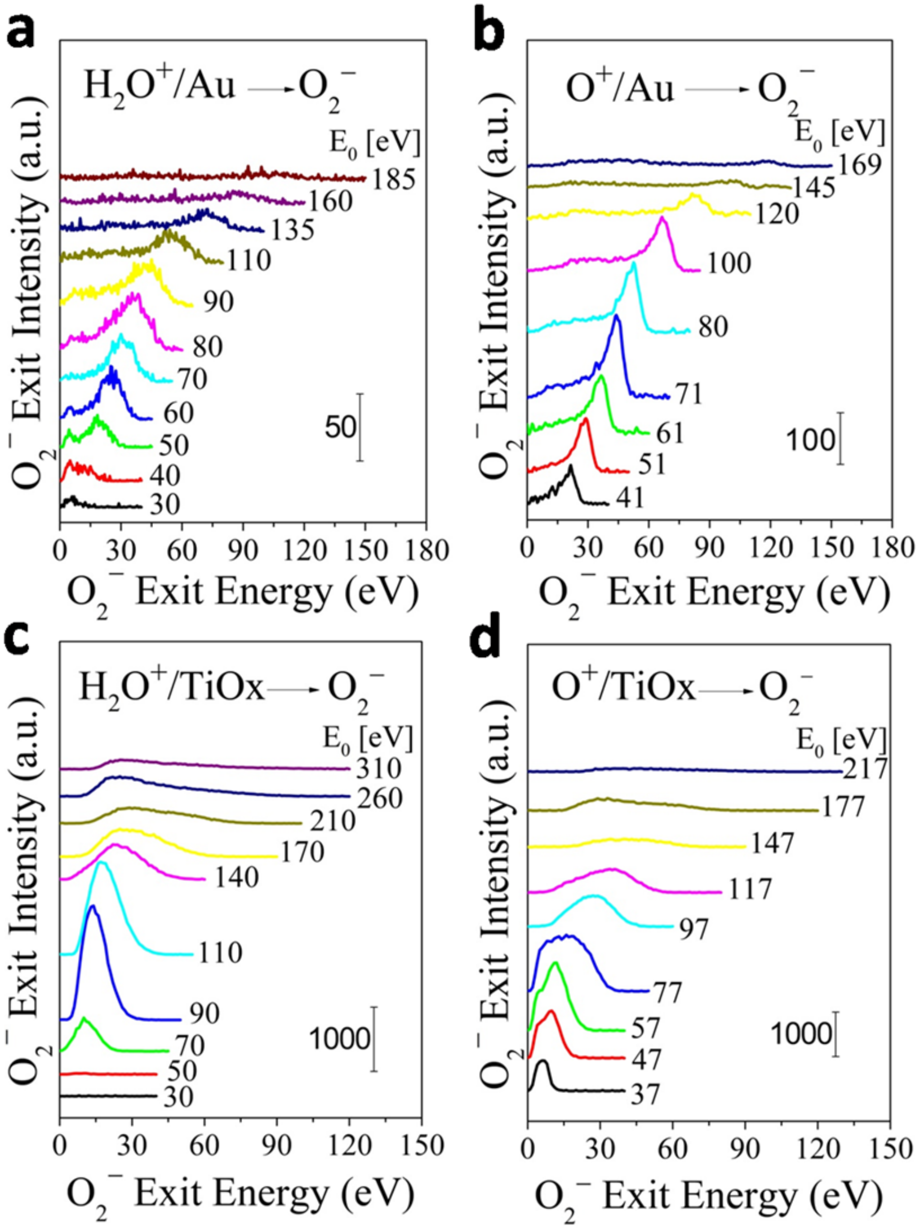

Supplementary Figure 12. O_2^- formation from H_2O^+ (and O^+) ion scattering on Au and on native titanium oxide. Energy distributions of O_2^- from (**a**) H_2O^+, (**b**) O^+ scattering on Au, (**c**) H_2O^+, and (**d**) O^+ scattering on native Ti oxide. The O_2^- formation is mainly due to the Eley-Rideal reaction between energetic H_2O^+ or O^+ ions and surface adsorbed O atoms. The surface O atoms on Au originate from the energetic incident ions via collision induced dissociation (H_2O^+) or trapping adsorption (O^+) after multiple bounces. Note that energetic O^+ can be readily produced in H_2O^+ or O_2^+ ion collisions with surfaces.

Supplementary Table 1. Estimation of surface sputtering contributions to O_2^- formation from isotopic dosing experiments. (**a**) H_2O^+/FeOy and (**b**) H_2O^+/SiOx.

a

$P(^{18}O_2)$ Torr	Relative ^{18}O coverage (θ)	$I(^{16}O^{16}O^-)$	$I(^{18}O^{16}O^-)$	$I(^{18}O^{18}O^-)$	Calculated sputtering $I(^{16}O^{16}O^-)$	Calculated sputtering $I(^{18}O^{16}O^-)$	Total sputtering	Total O_2^- formation	Sputtering Contribution at E_0=110 eV
5×10^{-8}	0.09345	66794	6885	98	9223	1901	11223	73777	15.2%
1×10^{-7}	0.13114	64898	9795	202	8868	2677	11746	74895	15.7%

b

$P(^{18}O_2)$ Torr	Relative ^{18}O coverage (θ)	$I(^{16}O^{16}O^-)$	$I(^{18}O^{16}O^-)$	$I(^{18}O^{18}O^-)$	Calculated sputtering $I(^{16}O^{16}O^-)$	Calculated sputtering $I(^{18}O^{16}O^-)$	Total sputtering	Total O_2^- formation	Sputtering Contribution at E_0=310 eV
1×10^{-7}	0.1219	34196	4747	452	23456	6512	30420	39395	77.2%
